# Anatomical and Physiological Researches on the Organ and Function of Hearing in Man and the Vertebrate Animals. To Which Is Added the History of the Nervous Plexus of the Tympanum

**Published:** 1839-07

**Authors:** 


### ART. IV.

1. *Recherches Anatomiques et Physiologiques sur l'Organe de l'Ouie et sur l'Audition dans l'Homme et les Animaux Vertébrés, &c.* Par G. BRESCHET, &c.

*Anatomical and Physiological Researches on the Organ and Function of Hearing in Man and the Vertebrate Animals. To which is added the History of the Nervous Plexus of the Tympanum.* By G. BRESCHET, Member of the Institute of France, &c. With 13 Plates.—*Paris*, 1836. 4to, pp. 295.

2. *Handbuch der theoretischen und praktischen Ohrenheilkunde.* Von Dr. CARL GUSTAV LINCKE, ausübendem Arzte und Wundarzte, akademischem Privat-docenten zu Leipzig, &c. Erster Band, *die Anatomie, Physiologie, und pathologische Anatomie des Gehörorgans. Mit fünf lithographirten Tafeln.—Leipzig*, 1837.

*Manual of Theoretical and Practical Ear-Medicine.* By Dr. CHARLES GUSTAVUS LINCKE, of Leipzig. Vol. I. *containing the Anatomy, Physiology, and Morbid Anatomy of the Organ of Hearing. With five lithographic Plates. — Leipzig*, 1837. 8vo, pp. 682.

3. *The Cyclopædia of Anatomy and Physiology.* Edited by R. B. TODD, M.D. F.R.S. &c. &c. Parts XIV. and XV., *Articles*, "Hearing, Organ of," by T. WHARTON JONES; and "Hearing," by Dr. R. B. TODD.—*London, August*, 1838, *and January*, 1839.

4. *A Treatise on the Structure, Economy, and Diseases of the Ear, being the Essay for which the Fothergillian Gold Medal was awarded by the Medical Society of London.* By GEORGE PILCHER, Lecturer on Anatomy and Surgery &c. *With* 14 *Lithographic Plates.—London*, 1838. 8vo, pp. 324.

5. *Handbuch der Physiologie des Menschen.* Von Dr. JOHANNES MÜLLER, &c.

*Manual of the Physiology of Man.* By Dr. JOHN MÜLLER, &c. Part II. of Vol. II., *containing* "The Senses." *Section Second*, "The Sense of Hearing."—*Coblentz*, 1838.

6. *Recherches Pratiques sur les Maladies de l'Oreille, et sur le Développement de l'Ouie et de la Parole chez les Sourds-Muets.* Première Partie : *Traité du Cathétérisme de la Trompe d'Eustachi, et de l'Emploi de l'Air Atmosphérique dans les Maladies de l'Oreille moyenne.* Par le Dr. Deleau, Jeune.

*Practical Researches on the Diseases of the Ear, and on the Development of Hearing and Speech in the Deaf and Dumb.* First Part: *A Treatise on Catheterism of the Eustachian Tube, and on the Employment of Atmospherical Air in the Diseases of the Middle Ear.* By Dr. Deleau, Junior. *With two Lithographic Plates.* —*Paris,* 1838. 8vo, pp. 431.

7. *Philocophus, or the Deaf and Dumb Man's Friend.* By J. B. (Bulwer,) surnamed the Chirosophus.—*London,* 1648.

The first book in the above list contains researches, which, we are informed by M. Breschet in his Introduction, were commenced in 1815, on the occasion of a *concours,* at the Faculty of Medicine of Paris, for the situation of *prosector.*

The results have been from time to time communicated in different memoirs to the Academy of Sciences; and though published in great part some five or six years ago, in the "Annales des Sciences Naturelles," they were only formally given to the world in 1836, first in the "Mémoires de l'Académie Royale de Médecine," (Tome cinquième, 3[e]. Fascicule,) and then in a separate form. We have devoted some attention to researches on the structure of the ear, and can therefore, with some confidence assert, that M. Breschet, in the work before us, has unravelled the intricacies of the labyrinth very successfully, and has considered its various parts in a manner more consonant, perhaps, with the principles of philosophical anatomy than had been done up to the time he wrote. The plates attached to the work are well executed, and in general highly illustrative of the verbal descriptions. This favorable opinion is nevertheless open to some exceptions, both of a general and specific nature. The specific we shall have occasion to notice in the course of this article. The principal of the general objections we have to make are; that there are numerous repetitions, whereby the memoir is extended over a greater space than was necessary; that M. B., though he has proposed a few sufficiently appropriate new names, tries to make too much of his nomenclature; and that, perhaps, he assumes too hastily as discoveries of his own certain points in the anatomy of the ear, which have certainly been noticed and investigated by others before him.

The work of Dr. Lincke contains a most complete body of information regarding the ear, in an anatomical, physiological, and morbid-anatomical point of view. In it the history of the subject is portrayed with candour and learning, and the descriptive part is at once minute and accurate. Dr. L. has, to be sure, contributed little if anything new of his own, but he has given us an admirable abstract of all that has been written on the subject up to the time he published. A second volume on the Diseases of the Ear was promised soon to follow this on the Anatomy and Physiology, but it has not yet appeared; nor is it likely, we are afraid, soon to appear in a separate form. The cause of this we suspect is, that the first has not been sufficiently patronised. If so, we are not altogether surprised at it; for excellent as it is, the first volume is not a production likely to be appreciated by or to come within the wants of many; and as to a second volume on the Diseases of the Ear, executed in the same spirit, though it might, together with the one before us, form a valuable *Otological Cyclopædia*, it would not, like Kramer's work, be the manual required by the practical surgeon. However, we are glad to say, that we shall not be entirely deprived of the results of Dr. Lincke's labours in *ear-medicine*,* as we find it announced that he is to supply the articles on the diseases of the ear for the Dictionary of Surgery, commenced by Professors Walther and Radius, of Leipzig, and the late Professor Jaeger, of Erlangen, and still continued by the two former. An article on hearing instruments, and another on the injection of the Eustachian tube, have already appeared.

The article on the organ of hearing, by Mr. Wharton Jones is, from the nature of the publication in which it appeared, necessarily very much condensed. The author has nevertheless contrived to give a very comprehensive account of the structure of the ear, including its development and irregular conditions. He has drawn a just physiological estimate of each of the component parts of the organ, and determined their importance, relations, and analogies, throughout the whole animal series. Though Mr. W. J.'s descriptions seem for the most part newly sketched, or verified from nature, he has not refrained from making good use of Breschet's work. He has sometimes adopted, sometimes corrected the views of the latter, and has borrowed the most expressive of his figures. As our views of the structure of the ear coincide for the most part with those of Mr. Wharton Jones, we shall avail ourselves largely of his labours in the composition of the present article.

The article "Hearing," by Dr. Todd, is a very judiciously composed summary of what is known or conjectured regarding the function of the several portions of the organ of hearing, including the principal acoustical experiments and observations bearing on the subject.

The second part of the second volume of Professor Müller's work having come to hand only as this article is going to press, we are not able to make use of it so far as we would wish. The section on the physiology of hearing manifests the same elaborate investigation which characterizes the other parts of this excellent and distinguished physiological treatise.

Mr. Pilcher's volume is the Essay for which the Fothergillian gold medal was awarded by the Medical Society of London last year. The subject of the ear is one which has often before engaged the attention of the Medical Society of London, as appears from the articles in different volumes of their "Memoirs." Among others, "Observations on Deafness from Affections of the Eustachian Tube," by James Sims, M.D., the President of the time, in volume first; and in volume third, "Case of

* Though strongly disliking the introduction of new words, particularly words borrowed from the modern languages, we are induced to tolerate this Germanic barbarism for the sake of conciseness and convenience.—Ed.

Original Deafness with the Appearances on Dissection," by J. Haighton; for this Mr. Haighton received a silver medal. The Medical Society of London therefore, in selecting the "Ear" as the subject of competition for the Fothergillian medal of last year, have worthily continued to follow up what appears to have been an object with the Society from an early period of its existence. And every one must admit that they could not have more "judiciously" (to use an expression in Mr. Pilcher's introduction) "fulfilled the benevolent intention of the enlightened and liberal physician who committed this important trust to their charge," than in the attempt to further a practical knowledge of ear-medicine. The proposing of an essay on the "Structure, Economy, and Diseases of the Ear" was certainly of all modes the one best calculated to promote a practical knowledge of the subject, provided care were taken that the essay selected as worthy of the prize, was at least on a level with the present state of knowledge on the subject. It remains to be shown, in the course of the present article, whether this has or has not been done, in awarding the prize to Mr. Pilcher's Essay.

M. Deleau's work can scarcely be considered as a new one, being principally made up of his former memoirs on the same subjects. The present volume, which contains 431 pages, is entirely devoted to the consideration of catheterism of the Eustachian tube, and the employment of injections of atmospherical air. With all due allowance for the explanations and counter-explanations of the first employer of any particular mode of treatment, and especially of such a mode of treatment, and for such affections too, as that proposed and employed by Deleau, it must be confessed that this is much greater space than was actually required. In Deleau's work, there is much to praise, but there is also much to blame. A desire to make things look more favorable than they really are too often breaks through.

To our list of recent works on diseases of the ear, we have added, more for its curiosity than anything else, the ancient small volume entitled "Philocophus, or the Deaf and Dumb Man's Friend." It is a quaint dissertation on the possibility of persons born deaf learning to speak and converse by the assistance of the eye alone. The text for the commentary is the history, related by Sir Kenelm Digby, of a young Spanish nobleman born deaf, but who yet, by the sharpness of his eye and the acuteness of his intellect, acquired the power of knowing, from the motions of the lips of the speaker, the words pronounced, and thus learned to speak.

Uninfluenced by any other desire than to convey to our readers information as to what knowledge we really possess of the ear and its diseases, we take the books before us to serve as the text for three sections on the subject. The first, on the Anatomy, the second, on the Physiology, and the third, on the Diseases of the Ear.

The analysis and comments we shall have occasion to make will, we believe, justify the opinion of each work which we have just expressed.

### I. ANATOMY OF THE EAR.

Of the history of the progress of discovery in the anatomy of the ear, Dr. Lincke gives a good sketch. He commences by showing that, as was to be expected, the knowledge of the ear possessed by the ancients did not extend beyond the external parts. The works of Erasistratus and Herophilus, the celebrated practical anatomists of antiquity, have not come down to us, but some idea of the extent of their acquaintance with the structure of the ear may perhaps be conceived, if we admit, which is probable, that the nomenclature of the parts of the ear employed by Rufus of Ephesus, in his work "De appellationibus partium corporis humani," written in the first century after Christ, was derived from Herophilus. The names to which we allude, *λόβος, ἕλιξ, ἀνθέλιξ, κόγχη, τράγος, ἀντίτραγος*, &c., indicate only a knowledge of the external parts of the ear, to designate which, indeed, they are still in use. From this it would seem that, up to the time of Rufus of Ephesus, the parts contained within the *os temporis* remained unknown, unless we can understand a passage in Celsus (lib. viii. cap. i.) as evidence to the contrary. Galen compared the internal ear to a labyrinth. He was, however, quite unacquainted with the connexions, situation, and direction of the cavities and passages in the petrous bone.

The real investigations and discoveries in the anatomy of the ear began at the end of the fifteenth century, and, as was natural, proceeded from the external to the internal parts.

"If we review," says Dr. Lincke, "what has been done in the different epochs from Vesalius to Casserius, from the latter to Duverney, from Valsalva to Scarpa, and from Soemmerring to the present time, it must be confessed that the knowledge of the structure of the ear has been enriched by the most gifted and acute anatomists; and it is but justice to say, that it is especially to Italians that we are indebted for the greatest number of contributions: to the active spirit of investigation of the German and also of the French anatomists of the present time, however, much praise is due, as it is by means of their accurate and fundamental investigations that the subject has been developed so perfectly as it now is." (*Lincke*, p. 26.)

M. Breschet, in the work before us, does not profess to consider the ear in any other than the mammiferous animals; he, however, refers, by way of illustration, to several points of its structure in the other classes.* Mr. Wharton Jones describes the human ear, and only makes use of occasional general illustrations, drawn from comparative anatomy. Dr. Lincke confines himself exclusively to the human ear: whilst Mr. Pilcher devotes his first chapter, consisting of six sections, to comparative anatomy.

In our analysis of the books on the anatomy of the ear, we shall follow the usual order of description, taking the external parts first and then the internal, which is the order followed by Mr. Pilcher, in his Anatomy of the Human Ear, and also by Dr. Lincke. By this means we shall not be obliged to introduce M. Breschet until his presence is actually required. We would wish it, however, to be understood, that we consider the order followed by Mr. Pilcher, in his "general observations,"—an order which has been carried out to its fullest extent by Mr. Wharton Jones,—viz. the internal parts first, and then the external, as being the more philosophical.

* He has composed other three monographies on the structure of the ear in birds, fishes, and reptiles, of which the first two are published. M. B. also promises to publish the history of the cavity of the tympanum in man and the mammifera, with that of the development of the various parts of the ear, as a sequel to the work before us.—Rev.

*In limine*, we have to remark, that the external ear is formed of *true cartilage*, covered by a *perichondrium*, and not of *fibro-cartilage*, as Mr. Pilcher (pp. 28 and 36) and Dr. Lincke (p. 70) say. At p. 39, Mr. P. says "the *fibro*-cartilage of the pinna is highly elastic, and is formed of several pieces, &c." The *true* cartilage of the pinna, we believe, is composed of but a single piece; fissured here and there, certainly, but presenting a continuity of substance throughout, and also with the cartilage of the auditory passage.

Concerning the membrana tympani, Mr. Pilcher remarks (p. 47), that "the positive existence of a proper membrane between the two reflexions" (of the lining membrane of the auditory passage on the one hand, and the mucous membrane of the tympanum on the other,) "is not yet determined." And again he says, near the bottom of the same page, "most dissectors allow that an independent fibrous membrane does exist, and that it is not merely a thickening of the mucous surfaces." All this is unnecessary doubt about a comparatively simple matter. Let us compare what is said on the subject by Lincke and Wharton Jones. This, by the by, is one of the few subjects on which Dr. Lincke offers anything original of his own.

"According to the investigations which I instituted on the subject, I recognize," says Lincke, "only three layers in the membrana tympani, one external, one middle, and one internal. The *external* is loosely connected, and is the blind end of the general integument of the inner surface of the auditory passage. The *middle layer* is a pretty strong, dense, fibrous, and elastic membrane, which may by maceration be separated into two lamellæ, the outer of which is much more delicate and finer than the inner. It is, as I suppose, formed by an intimate incorporation of the periosteum of the tympanic cavity, and the osseous auditory passage, and is not immediately fitted into the tympanic ring, but takes its origin directly from a proportionally very thick, firm, and ligamentous ring, which partly lies in the *groove* for the membrana tympani, and is very intimately and closely connected with it. The two lamellæ are so closely connected with this ring, that I have not succeeded, after weeks of maceration, completely to separate them from it. The muscular fibres, first described by Home, and admitted by Sprengel, Lenhossek, Meckel, Buchannan, and other anatomists and physiologists, I have not been able, even with the assistance of the microscope, to perceive." (*Lincke*, p. 94.)

"As regards the composition of the membrana tympani," says Mr. Wharton Jones, "it consists of a proper membrane and two borrowed layers, &c." Concerning the nature of the external borrowed layer, we think neither Lincke nor Wharton Jones are sufficiently explicit. We have seen above that the former calls it "the blind end of the general integument of the auditory passage." The latter describes it as "a delicate continuation in the form of a blind end of the lining of the auditory passage," But, again he says, in speaking of its connexion with the proper membrane, "it readily separates from it by putrefaction, and can be drawn out along with the rest of the *epidermis* of the external auditory passage in a cul-de-sac." The fact is, the layer, so easily separated from the external surface of the membrana tympani, is merely epidermis. This being so, we must have, also, in the composition of the membrana tympani, some sort of chorion. The fibrous layer of the membrana tympani has in its structure a continuation of the elements of the chorion of the skin of the auditory passage. This remains inseparably incorporated, whilst the epidermis comes away by putrefaction. We agree, in other respects, with the following remarks regarding the "structure of the proper membrane."

"The proper membrane can be divided into two layers, an outer thin one, consisting of radiating fibres, and an inner thicker layer, which is less distinctly fibrous, though when torn it does indicate a fibrous disposition, and that in a direction opposite to the former. The radiating fibres run from its circumference towards the centre, to be fixed to the handle of the malleus, along its whole extent. Towards the centre they become stronger, and being of course more aggregated, the layer which they compose is thicker and more compact in the centre than towards its circumference. The fibres which cross the radiating ones are also more aggregated at the centre. They run parallel with the handle of the malleus, and turn round its extremity. At the circumference of the proper membrane there is a thick, firm, ligamentous, or cartilaginous ring, which is fixed in the groove of the bone. This ligamentous ring appears to be formed by an aggregation of the circular fibres interwoven with the peripheral extremities of the radiating ones. The part of the membrana tympani, midway between its centre and circumference, is the thinnest. The radiating fibres have been supposed to be muscular by Sir Everard Home and others, but this has not been confirmed by microscopical examination." (*Wharton Jones, Cyclopædia*, &c. No. xiv., p. 545.)

The following is the same writer's account of the connexion of the malleus with the membrana tympani:

"That edge or ridge (on the handle of the malleus) which is continued down from the short process is turned outwards, and corresponds to the membrana tympani; into it, indeed, along its whole extent, the central extremities of the radiating fibres of that membrane are inserted. The extremity of the handle of the malleus, which is curved forwards and outwards, is compressed, but in a direction contrary to the rest of the handle; so that one of the flat surfaces, that corresponding to the outer ridge of the rest of the handle, is connected with the membrana tympani at a point below its centre, and nearer its anterior edge. It is at this point that the bottom of the concavity is, which the membrana tympani presents externally. At its upper part, the membrana tympani is pushed outwards by the short process of the malleus, which projects towards the auditory passage." (*Ib.* p. 547.)

The membrana tympani is a part of so much practical importance, that we make no apology for dwelling on it at this considerable length. Mr. Wharton Jones has given an exposition of the subject, useful alike in a surgical and in a physiological point of view.

"Mr. Shrapnell describes, at the anterior and superior part of the membrana tympani, above the short process of the malleus and its suspensory ligament, and where the groove in the bone is deficient, a flaccid tissue, composed of irregularly arranged fibres, to which he gives the name of *membrana flaccida*, in opposition to the rest of the membrana tympani, which he calls *membrana tensa*. This flaccid tissue is more developed in some of the lower animals, the sheep and hare, for instance, than in man, and can be readily made to bulge out towards the auditory passage, by blowing air into the Eustachian tube. But we cannot look upon it with Mr. Shrapnell, as properly forming any part of the membrana tympani. It is merely a mass of dense reddish vascular cellular tissue, surrounding the neck of the malleus, and continuous with a similar tissue, found under the lining integument of the upper wall of the osseous auditory passage. It is this same tissue which has been described as a muscle and sometimes as a ligament." (*Ib.* p. 546.)

It is of the greatest importance to the surgeon to know well the natural appearance which the membrana tympani presents in the living body.

"It is situated at the bottom of the external auditory passage, between which and the cavity of the tympanum it is interposed, like a partition. It is a thin, semitransparent, glistening, dry-looking membrane. Its shape is an oval, truncated at one extremity, the upper. Rather more than the upper half of its vertical diameter is traversed by the handle of the malleus, *which*, when the membrane is examined, in the living subject, by means of the speculum auris, *appears directed from above downwards and backwards.*" (*Cyclopædia*, &c., p. 545.)

In direct opposition to this last assertion, Mr. Pilcher informs us, at p. 53, that the handle of the malleus descends from the cervix and processus brevis, being a little inclined forwards, &c.

We would recommend to our young friends to exercise themselves in examining the auditory passage and membrana tympani in the living person, and to note well the ordinary position and direction of the handle of the malleus. One or two examinations will, we think, be sufficient to inform them whether, in regard to the upright position of the body, the handle of the malleus be directed backwards or forwards.

We now turn to another part of great importance, in the surgical anatomy of the ear, viz. the Eustachian tube. The practical points to be considered, in regard to this, are its length, its width at different places, and the size and position of its guttural orifice.

*Length.* The length of the Eustachian tube is given, by Lincke, at one inch seven or eight lines, Parisian measure, the osseous portion of the tube being seven or eight lines, (p. 147,) and the cartilaginous portion about one inch. (p. 149.) Wharton Jones states the length of the Eustachian tube at one and a half inch, (p. 546,) the osseous portion being half an inch, (p. 546,) and the cartilaginous and membranous portion about one inch. (p. 550.) Pilcher says (p. 51) the Eustachian tube is upwards of one and a quarter inch long.

*Width.* In regard to the width of the Eustachian tube, it is known that its guttural orifice is wider than that by which it opens into the tympanum. It is also acknowledged that the narrowest part is where the bony and cartilaginous portions join, but there is much difference of opinion as to the exact degree of width of the narrowest part. Mr. Pilcher describes the tube as being slightly contracted from the "tympanum to the cartilaginous portion," (p. 51;) and delineates, in plate 9, (the different figures of which plate, we are given to understand, are " faithful representations of recent dissections of the human ear,") the narrowest part as being about one tenth of an inch. Lincke says on the point, (p. 147,) from the tympanum, the Eustachian tube gradually contracts, and is, as Valsalva has already correctly asserted, at the end of the bony portion, from three quarters to one line in its largest diameter.

Its transverse diameter, on the contrary, continues Lincke, amounts, at its opening into the tympanum, to about one line; it then becomes gradually smaller, and measures, at the end of the osseous portion, half a line. Sometimes this transverse diameter is still less, especially where the canal is yet lined by its membrane. In consequence of this, the caliber of the canal is at this place so narrow, as scarcely to admit the passage of a thin probe. Lincke further adds, " the diameter of a quarter of a line, at the narrowest part, as given by Kramer, is too small." We would remark, in regard to the criticism of Kramer by Lincke, that Kramer says the width, at the narrowest part of the tube, is one thirty-second of an inch or a quarter of a line. Kramer's error consists merely in speaking of one thirty-second of an inch as equal to one quarter of a line, whereas, the thirty-second of a Paris inch is rather more than one third of a Paris line.*

The following is Mr. Wharton Jones's observations on this subject:

"Its (the osseous portion of the Eustachian tube) caliber contracts in its course forwards, and is compressed from without and below inwards and upwards. In the dry bone it is wide enough to admit the end of a quill stripped of its feathery part, about one twelfth of an inch thick. In the recent state, when lined by its mucous membrane, it is very much narrower." (*Cyclopædia*, p. 549.)

"The point of junction (between the bony and cartilaginous portions of the tube) is the narrowest part of the tube; in the recent state about one thirtieth of an inch in diameter, just sufficient to admit a small probe." (*Ib.* p. 550.)

We thus see that Kramer and Wharton Jones nearly coincide; for the Paris inch, which we suppose Kramer uses, is somewhat longer than the English inch, according to which the English author calculates.

*The size and position of the guttural orifice* are the most important points in the surgical anatomy of the Eustachian tube. On the subject, Lincke says, (p. 148,) that he has not found Kramer's account of it correct. The following is Mr. Wharton Jones's account of it:

"The mouth of the Eustachian tube in the throat forms an oval-shaped fissure, about three-eighths of an inch long, bounded anteriorly and posteriorly by prominent swollen edges. The fissure is directed obliquely, from above downwards and from before backwards, and is situated at the upper and lateral part of the pharynx, behind the soft palate. In reference to the nasal passage, my observation agrees with that of Kramer, that the *lower angle* of the guttural orifice of the Eustachian tube *lies a very little deeper than the horizontal line of the lowest meatus, whilst the upper angle is a little deeper than the horizontal line of the middle meatus.*" (*Cyclopædia*, p. 550.)

Still another point in the anatomy of the ear, of consequence in a practical point of view, is the nature of the membrane which lines the cavity of the tympanum. Mr. Wharton Jones describes it as follows:

"The lining membrane of the cavity of the tympanum is in continuity with the mucous membrane of the throat, through the Eustachian tube. Extremely delicate, and in some parts very vascular, it is not merely a mucous membrane, but is theoretically a combination of periosteum and mucous membrane, being what Bichat called fibro-mucous. It invests all the elevations and depressions observed on the walls of the tympanum, and extends into the mastoid cells. The outer layer of the membrane of the fenestra rotunda, *membrana tympani secundaria*, is a continuation of it. The base of the stapes is fixed by its circumference to the outer edge of the groove, which encircles the vestibular fenestra, by a membrane or ligament. The lining membrane of the vestibule, continued over the base of the stapes from within, also invests the inner surface of this annular ligament, whilst the outer surface of it is covered by the membrane lining the tympanum, as it is reflexed on the stapes. The membrane lining the tympanum invests the small bones and the tendons of their muscles, where they run free in the cavity. A fold of it fills up the space, bounded by the crura and base of the stapes. The chorda tympani, also, in its passage across the tympanum, is enveloped by it. Lastly, it forms the inner borrowed layer of the membrana tympani, covering and adhering closely to the handle of the malleus." (*Cyclopædia*, p. 549.)

* Lines, French measure, are very often mentioned, in English books, with the most vague signification; and many authors seem to prefer making their calculations in French lines and half lines, without, however, any clear notion of the length of a line, to using tenths and twentieths of an English inch. Mr. Ross, the optician, in Regent street, makes a scale of lines and half lines, French measure, and of tenths and twentieths of an inch English measure. We find the scale very useful, and would recommend it to our friends.—REV.

Ruysch was the first who unequivocally demonstrated, by his injections, that the ossicles are invested by a periosteum. Mr. Pilcher, speaking of the ligaments of the tympanic ossicles, remarks,

"H. Cloquet says the ossicles are unfurnished with ligaments, and are held together merely by the common mucous membrane of the tympanum, which he seems to believe also constitutes the only periosteum they possess. . . . . The anatomists of this country, on the contrary, have usually described ligaments; and there can exist but little doubt that some of the folds of the membrane are so strengthened as to merit this distinction; of these particularly may be mentioned the *triangular ligament*, occupying the space between the crura and base of the stapes, and the *ligament* of the short crus of the incus passing to the edge of the mastoid cells." (*Pilcher*, p. 55.)

"So delicate and thin," continues Mr. P., "are the other ligamentous attachments, that a doubt may be justified as to the existence of more than mucous membrane." Now, we are of opinion, that doubt is not justified, for surely our knowledge of tissues is now sufficiently far advanced to enable us to distinguish ligament from mucous membrane. "There are, however," adds Mr. P., "usually enumerated, 1, a ligament fixing the manubrium and short process of the malleus to the membrana tympani; it is very certain this process of bone is placed between the mucous and proper membranes, and its union appears also to be strengthened by cellular tissue." There is no ligament fixing the handle of the malleus to the membrana tympani. It is the fibres of the proper layer of the membrana tympani itself that are inserted into the handle and short process of the malleus, as we have already seen.

Before leaving this part of our subject, we must here notice certain circumstances relating to the accessory parts of the apparatus of hearing; and in doing so, we shall avail ourselves of the accurate description of Mr. Wharton Jones.

"In some animals, such as the mole, the squirrel, the guinea-pig, the marmot, &c., there is an osseous canal, like a bar of bone, extending over the vestibular fenestra, and running through between the crura of the stapes. This was first observed by Sir Anthony Carlisle in the marmot and guinea-pig, who describes it as 'an osseous bolt to rivet it (the stapes) to its situation.' The canal is for the passage of an artery and nerve, which in some other animals are unprovided with an osseous canal in their course through the stapes. The artery running through the stapes was observed about ten years ago by Professor Otto, in hybernating animals; but Professor Hyrtl, of Prague, has shown that the artery is by no means peculiar to those animals, as it does not occur in all, and as it occurs in animals which do not hybernate.

"Mr. Shrapnell describes in the human ear an artery, accompanied by a nerve passing through the membrane, which fills up the space between the arms of the stapes. Mr. Shrapnell was led to this observation from what he had seen in the rat, viz., a nerve and artery passing through the stapes, and supported by a minute channel of bone. Professor Hyrtl has more recently described three modes of distribution of the arteries in man, which he has met with, analogous to the artery running through the stapes in the animals above mentioned." (*Cyclopædia*, p. 556.)

Two or three years ago, Hyrtl published (in Müller's Archives, if we remember aright) a short notice of the artery running through the canal of bone, and thought he had been the first to notice it. Professor Otto, however, as is seen from the above extract, had described it ten years before; and our own countryman, Mr. Shrapnell, had described the substance of what Hyrtl, several years subsequently, brought forward in the Oesterreichische Jahrbücher. These papers of Mr. Shrapnell, in the Medical Gazette, (vol. x., 1832,) though defective in several respects, contain some very ingenious observations, and do not appear, hitherto, to have been so well known and appreciated as they deserve.

The Labyrinth now falling to be considered, we take up Breschet's work, and shall follow the order of it in our remarks. We had written a very full abstract of this part of Breschet's volume, but now find we have not room for more than notices of points prominent either in regard to their novelty or importance.

Breschet only notices slightly the osseous labyrinth. It is the membraneous labyrinth he dwells most on, and in doing so we think he has done service, by raising to a more prominent position a part of the ear, which, to judge from the way it is usually handled in our anatomical books, one would suppose to be, instead of the most important, as it really is, but a very unessential part of the organ of hearing.

*Osseous labyrinth.* The osseous labyrinth in man and the higher animals consists of three parts: the vestibule, semicircular canals, and cochlea; all of which cavities communicate. To the aggregate of them Breschet gives the name of *labyrinthic cavity.* The labyrinthic cavity is lined by a delicate membrane of a serous or fibro-serous nature. The membraneous labyrinth is not to be confounded with this pellicle. By a particular arrangement the lining membrane of the labyrinthic cavity completes the spiral septum of the cochlea, and contributes to form the membranes closing the vestibular and cochlear fenestræ. A very important point to be remembered in the anatomy of the labyrinth, and which is insisted on by Breschet, but particularly by Wharton Jones, is that the cochlea belongs to the system of the osseous labyrinth; that it is a compartment of the labyrinthic cavity; that it contains nothing of the membraneous labyrinth, only liquid of Cotugno.

"The cochlea," says Mr. Pilcher, in his General Observations, "is very frequently wanting, the canals (semicircular) being then the only part of the second division added to the vestibule, &c." And again, "In structure the cochlea corresponds to the rest of the labyrinth, in being a cartilaginous or bony canal, lined by a vascular membrane, which contains the liquor cotunni; but it is not usual to find a second membraneous tube within this fluid, the nerve being expanded upon the vascular lining." (p. 16.)

This does not exhibit a correct general view of the nature of the cochlea. The cochlea is, in fact, an appendage of the osseous or cartilaginous labyrinth, and has nothing in common with the membraneous labyrinth. The osseous labyrinth may be more or less perfect; the cochlea may exist or not, and yet the membraneous labyrinth (comprising the membraneous vestibular sacs, ampullæ, and semicircular tubes,) be even very highly developed, as in fishes. The lining vascular membrane of the cochlea is the same as the lining pellicle of the osseous or cartilaginous vestibule, but has nothing to do with the membraneous labyrinth. The nerve of the cochlea is expanded *outside* the vascular lining membrane. The membraneous labyrinth being situated inside the vascular lining pellicle of the bony or cartilaginous vestibule and semicircular canals, of course the vestibular nerve pierces that pellicle to arrive at the membraneous labyrinth.

It thus appears that a distinction ought to be kept in view between the labyrinthic cavity and the membraneous labyrinth, as without this there can be no correct general conception of the internal ear, and we are constantly liable to run into error when we attempt to estimate the nature of the parts of the ear of different animals. An example of this error is found in Mr. Pilcher's work, (p. 20), where he says, "As we examine the apparatus in the advancing series, we find, probably, a rudimentary cochlea in the sacculus of fish." On the contrary, the sacculus vestibuli of fish belongs to the system of the membraneous labyrinth, whilst the cochlea, when it does exist, forms a part of the system of the cartilaginous or bony labyrinth.

A point in the cochlea worthy of notice is the hole by which the two scalæ communicate at the apex; this hole Breschet calls *helicotrema.* The circumference of it is formed, two thirds by the free concave margin of the hamulus of the bony spiral lamina, the rest by the membraneous zone of the spiral lamina. In the recent state it is through the helicotrema only that the tympanic scala communicates with the rest of the labyrinthic cavity.

The aqueducts are appendages of the osseous labyrinth. Much difference of opinion exists in regard to them.

There are two humours contained in the labyrinth: one which belongs to the cavity of the osseous labyrinth, and which appears to be the secretion of its lining membrane; another, contained within the membraneous labyrinth. The former, commonly known by the name of liquid of Cotugno, is called by De Blainville and Breschet *perilymph.* The latter, Breschet calls *endolymph.* It is an old notion, and one which, in our time, has found for defenders Brugnone and Ribes, that there is an *aer ingenitus* in the labyrinth. "On this point," says Breschet, "the observations of P. F. Meckel appear to us quite conclusive, as to the non-existence of air in the labyrinth; and they are in perfect accordance with our own observations and those of Cotugno." In 1825, however, when, in conjunction with Jourdan, he published the translation of J. F. Meckel's Anatomy, Breschet appears, from a note in vol. iii. p. 189, not to have adopted the opinions of P. F. Meckel and Cotugno, but to have agreed with Ribes.

The liquid of the osseous labyrinth, or perilymph, occupies, in the vestibule and semicircular canals, all the space not taken up by the middle sinus, the saccule, and semicircular tubes. It fills, moreover, the cochlea, and as all these parts communicate, it is the same humour in each.

Some anatomists, in taking for the prototype of their descriptions the organ of hearing in fishes, have fallen into an error, inasmuch as not considering that in most fishes the membraneous labyrinth constitutes almost exclusively the auditory apparatus, and that what of the labyrinthic cavity does exist communicates freely with the cranial cavity, they have confounded the liquid of the membraneous labyrinth with that of the osseous labyrinth in the higher animals. "It is the encephalic liquid itself,"

Breschet justly remarks, "which in the greater number of fishes we must compare with the perilymph."

"Cotugno and Meckel," says Wharton Jones, "supposed that the aqueducts were a sort of diverticula, or cavities which served to let off the superabundant perilymph, when necessary, during the act of hearing. This opinion is, however, now-a-days, very much questioned; and several anatomists, Brugnone, Ribes, Breschet, &c. refuse to those aqueducts the uses which Cotugno assigned them, and consider them merely as canals destined for the passage of blood-vessels. Although they may be insignificant in a physiological point of view, still, if the description I have given of them be correct, they must be considered as something more than mere canals for the transmission of vessels. The constancy of the aqueducts, moreover, is another argument against their being mere vascular canals.

"Breschet (and, in Hildebrandt's *Anatomie*, by Weber, the same idea is concisely expressed,) explains the mode of formation of the aqueducts, by supposing that at first the labyrinthic cavity is nothing but a sac formed by a prolongation of the dura mater in the same way as the tunica vaginalis is of the peritoneum; that, as development proceeds, the tube of communication between the labyrinthic sac of the dura mater and general cavity of the dura mater is gradually contracted and elongated; and that, as ossification extends, the tube becomes surrounded by osseous substance, and presents itself under the appearance of an aqueduct.

"This view (says Breschet,) is rendered probable; for, in many fishes, the labyrinthic cavity forms one with that of the cranium. And if, in these animals, a prolongation of the walls of the cranium tended to separate the brain from the ear, there would result a small canal establishing a communication between the two cavities, and this canal would be nothing but an aqueduct." "According to this view," continues Wharton Jones, "the lining membrane of the labyrinthic cavity may be considered as a continuation of the arachnoideal layer of the dura mater, perhaps of the dura mater also." (*Cyclopædia*, p. 536.)

*Membraneous labyrinth.* As a type of the soft parts of the internal ear, Breschet adopts the membraneous labyrinth of the frog-fish (lophius piscatorius), in which all the component parts exist in a high degree of development. The membraneous labyrinth of fishes resembles, fundamentally, that of the mammifera. The component parts of the membraneous labyrinth are the common sinus, the membraneous ampullæ, with the semicircular tubes, and the sacculus. These constitute an apparatus, the counterpart of the vestibule, ampullary sinuses, and semicircular canals of the osseous labyrinth, in which they are contained. No part of the apparatus of the membraneous labyrinth is contained in the cochlea. The common sinus and saccule are fixed to the inner wall of the vestibule by the nervous filaments which they receive. Towards the outer wall of the vestibular compartment of the labyrinthic cavity, they are not in contact with the base of the stapes, but are separated from it by the intervention of the perilymph. This circumstance, first distinctly pointed out by Scarpa, is very particularly insisted on by Breschet, to show that it is only by the intermedium of the perilymph that the movements of the stapes can have any impression on the nervous expansion of the membraneous labyrinth.

It will be in the remembrance of our readers that we have already referred, in the article in our Twelfth Number, to the minute structure of the auditory nerve, and its expansions in the membraneous labyrinth. We shall not, therefore, recur to the subject.

"In the sheep, hare, rabbit, &c. the walls of the membraneous labyrinth present patches of black pigment, a circumstance noticed by Scarpa, Comparetti, and

Breschet. Before I knew of the observations of these anatomists I had myself observed the fact. I was not, however, led to the discovery of it by accident, but, being engaged in researches on the pigment of the eye, and considering the analogy which the organs of sense bear to each other in their general anatomical structure, I was curious to know whether pigment did not exist also in the ear. Examination proved to me that it did; for I found, as Scarpa and Comparetti had previously noticed, pigment deposited in the form of small black spots, in the membraneous parts of the labyrinth in different mammifera. In some I have found a distinct cellulo-vascular layer of a black or brown colour, forming the outer surface of the membraneous labyrinth. And, contrary to what Breschet asserts, I have found pigment in the membraneous labyrinth of the human ear also. It appears, especially on the ampullæ, under the form of a slight but perfectly distinct brown tinge, similar to what is seen around the ciliary processes in the eyes of albinoes."

(*Wharton Jones, Cyclopædia*, p. 538.)

We have noticed the humour of the osseous labyrinth. The membraneous labyrinth contains, also, a limpid humour, which Breschet calls *endolymph*, in contradistinction to *perilymph*. The humour of the membraneous labyrinth was first distinctly pointed out by Scarpa.

Contained in the endolymph are small masses of calcareous matter: in some animals solid, in others pulverulent. In the mammifera the chalky matter is in small quantity, and pulverulent; in fishes (with the exception of the chondropterygenous, with fixed gills,) it presents itself in the form of solid, and sometimes large masses.

"These calcareous masses," says Wharton Jones, "are best known in osseous fishes, in which they are hard but brittle bodies of a determinate shape. In these animals, indeed, they have been erroneously considered as analogous to the tympanic ossicles of the higher vertebrata. MM. Breschet and Huschke have lately called particular attention to the subject, and have described masses of calcareous matter in the ear of reptiles, birds, and mammifera. Scarpa and Comparetti had observed them in the human ear, without, however, detecting their nature. But they had been unequivocally noticed before by De Blainville; and, previously to the first publication of Breschet's papers on the ear, in the 'Annales des Sciences Naturelles,' I had also studied them throughout the animal series." *Cyclopædia*, p. 539.)

"For the sake of greater precision and perspicuity in our descriptions," says M. Breschet, "we have given the name of Otoconies (from *οὖς, ωτὸς*, *auris*, and *κόνις, κόνεος*, *pulvis*,) to the concretions which are pulverulent; and the name of Otolites (from *οὖς* and *λίθος*, *lapis*,) to the solid stony concretions."

The following extract from Wharton Jones, is a summary of all that is known of importance regarding the calcareous matter of the ear.

"In the ear of man and the mammifera in general, there are two masses of calcareous matter; one in the common sinus and the other in the saccule. According to Huschke and Barruel, they are composed of mucus, carbonate and phosphate of lime, and some animal matter. They are said to be more distinct in the fœtus than in the adult. From my own observations, I should say that they exist in the human adult as distinctly as in the fœtus. Concretions are never found in the ampullæ, or semicircular tubes, either in man or any of the lower animals. Examined in man and the mammifera, the concretions are suspended in the endolymph, and correspond to the points of the common sinus and saccule, where the nervous filaments are implanted. The grains composing the calcareous mass are held together by a soft mucous tissue. Huschke describes the grains as crystalline, small six-sided columns, pointed at the ends, with three surfaces; they appear to me, under the microscope, to have an oval form, more or less elongated, in man and the mammifera, passing into a spindle shape in birds and reptiles; and, though transparent, they do not present any very decided crystalline form. . . . . . .

"The grains of the ear are of different sizes. Of a mass which I removed from the ear of a middle-aged man, the greatest number had their longest diameter equal to that of the globules of the human blood, that is, about the three-thousandth part of an inch.

"There is found in the cochlea of birds a mass of calcareous matter; Breschet says he has found, in cochleæ of the human fœtus, which had been dried but not macerated, small masses of cretaceous matter, deposited near the summit of the cochlea; and Huschke once found, in the fluid of the cochlea of a child, a collection of microscopical crystals." (*Cyclopædia*, p. 539.)

On turning to Mr. Pilcher's work, the most recent of all those at the head of this article, to see if he had adduced any additional information on the subject, we were surprised to read at p. 70, that Mr. P. has not been able to see the *otoconies* at all, but he *grants* that, "as they are so generally met with in the lower animals, *yet most probably not universally in mammalia*, it is possible that *something of the kind* may exist, even in the human subject; and it is certain that a whitish matter is often found upon the surface of the membrane." Again, at p. 146, Mr. P. says, "Itard has described a calcareous body in the vestibule, as a mal-deposit producing deafness. It is possible that this appearance may be the otolithe of Breschet, although we have doubted its existence in the human ear. If it should occur as a malformation, it affords another example of the occasional approximation in peculiar structures of man to those of the lower animals." Allowing that Mr. Pilcher never succeeded in demonstrating, to his own satisfaction, the existence of the *otoconies*, we own we are surprised to find him stating his opinion thus strongly, after what has been written by Breschet, Huschke, and Wharton Jones.

### II. PHYSIOLOGY OF THE EAR.

DR. LINCKE gives an historical sketch of the progress of the physiology of the ear in like manner as he did of its anatomy. "The new ideas and remarks," he says, "incidentally made by Berengarius of Carpi, Vesalius, Ingrassias, Colombus, Fallopius, and Eustachius, on the function of the parts discovered by them in the ear, are not, indeed, without interest, but are in general so unimportant and unsatisfactory, that it is not worth the pains to notice them in detail." (p. 265.) Volcher Koyter was the first who attempted, by collecting and arranging all that was known on the subject, to give a complete explanation of the mechanism of hearing; and this he did in a way pretty satisfactory, considering the time. He left his predecessors and contemporaries far behind; and though his notions regarding the proper act of hearing be very defective, still his explanation of the function of the accessory parts of the ear is, for the most part, what is still admitted as by many physiologists correct. Fabricius ab Aquapendente and Casserius introduced some modifications into the views of Koyter; and most of the contemporaries, and many of the successors of these three men, either wholly coincided in their opinions or differed but little from them. Among those who, in the last half of the seventeenth century, strove to enlarge our knowledge of the physiology of hearing, Molinetto, Willis, Schelhammer, and Duverney, deserve to be named.

A peculiar subtile, innate air, *aer ingenitus*, supposed to be contained in the ear, played a most important part in all the explanations of hearing, given by the earlier anatomists and physiologists, Democritus, Diogenes, Appolonia, Aristotle, Koyter, &c. The existence of this *aer ingenitus* had been already called in question by Bauhin, but it is to Schelhammer that science is indebted for having first forcibly combated the opinion.

Duverney, in the second part of his treatise on the ear, discussed what was known of the physiology of hearing with accuracy and perspicuity, and made several new observations of his own in regard to the membrana tympani and labyrinth. It was not, however, until 1757 that anything like a correct physiology of hearing existed. The author of the new light shed on the subject was Dominico Cotugno. He gave his voice in favour of the correctness of Schelhammer's assertions as to the non-existence of an *aer ingenitus*, and established incontrovertibly the presence of a liquid in the labyrinth. Viewing it as performing some office in the exercise of hearing, he laid the foundation for a new theory of that function—a theory which, with some modifications, has been generally adopted by all physiologists since.

The study of the physiology of hearing necessarily embraces the further history of the subject. It must be confessed that, notwithstanding all that has been done, we do not yet satisfactorily comprehend the whole mechanism of the act of hearing.

"As the apparatus of vision," says Wharton Jones, "naturally admits of being divided into two parts, viz., the eyeball and its appendages, so we can distinguish in the apparatus of hearing a fundamental organ, and parts accessory to the perfect performance of its function. The fundamental organ of hearing is what is commonly called the internal ear, or, from the complexity of its structure, the *labyrinth*. The accessory organs consist of the middle ear or tympanum and external ear." (p. 529.)

One part of the eyeball transmits and concentrates the rays of light, which, falling on the retina, produce an impression, which is propagated along the optic nerve to the brain. It is the mind that perceives the impression and draws inferences from it. We are not to expect to find in the labyrinth an organ of sense doing more in regard to sound than the eyeball does in regard to its object. In short, we must not expect to perform with the eye or the ear what is evidently the function of the brain. "The eye of a painter" and "the ear of a musician" are merely figurative expressions. It is the mind that perceives colour and proportion in the one, and harmony of sound in the other. If this be the case, then it is evident that there is as little use for a musical part in the organ of hearing as there is for a painting part in the eye. To distinguish clearly what share the mind has and what share the ear, in the act of hearing, must be a great means of arriving at accurate notions on the subject.

*Use of the external ear and auditory passage.* Some have asserted that the external ear is of no use in hearing; others again endeavour to make it out to be an almost indispensable part of the ear. As in many other cases, truth will be found between these two extreme opinions. The external ear collects the undulations of sound, and reflects them into the auditory passage, by which they are transmitted to the middle ear. As the auditory passage first becomes narrower towards the middle, and then increases in width to the membrana tympani, sound will first be concentrated and then moderated in its progress. The external ear and auditory passage are, in fact, as Dr. Todd observes, a hearing trumpet. Savart's experiments to ascertain how far the external ear and auditory canal serve to increase the vibrations of the tympanic membrane, though good, have this defect, that a conical tube was used instead of one contracted in the middle, and dilated at the extremities, as the auditory passage is.

Does the external ear assist us in judging of the direction of sound, as Treviranus supposes? It can only do so indirectly, by receiving, (when the head has been properly directed,) and then conveying to the internal ear the sound, and all the different modifications produced on it in its passage through the interposed medium or media; then, according to the impression on the nervous expansion of the membraneous labyrinth, will the mind, assisted by past experience, judge of the direction of the sound. Ventriloquism, by imitating the peculiarities which characterize sound, issuing from this or that direction or place, produces its illusive effects.

The ear-wax appears to be of use in hearing only by maintaining the lining integument of the auditory passage in its proper organic condition. The secretion of the Meibomian glands, &c. does not actually make the eyeball see better, but it renders the eyelids more capable of all those minute and delicate actions which assist in perfecting the act of vision.

*Use of the membrana tympani.* "Besides the undeniable use of the membrana tympani, in protecting the middle ear against external injury, it has the still more important one of being thrown into vibration by the undulations of sound coming along the auditory passage, and thus contributes to the finer tones. . . . . Cases of perforation or destruction of the membrana tympani, only prove that the membrane is not indispensably necessary for hearing; but that in persons in whom it is either partially or wholly destroyed by ulceration, fine and distinct hearing undergoes a modification, and admits of no comparison with the ordinary hearing power. (*Lincke*, p. 454.)

In consequence of the connexion of the membrana tympani with one end of the chain of ossicles, the tension of it is capable of being varied by the action of the muscles of the ossicles. The membrane of the fenestra ovalis being connected with the other end of the chain, the tension of it is varied at the same time. Hence, there must be some relation between the state of tension of the soft parts within the labyrinth, and that of the membrana tympani. According to the state of tension of the membrana tympani, so will the vibrations of sound be communicated to the labyrinth. Professor Müller is of opinion that increased tension of the membrana tympani causes dulness of hearing. "Not unfrequently," says he (p. 438), "deaf persons have lost the power of hearing the graver tones only, whilst they retain the faculty of perceiving acute though low tones. A deaf colleague of mine hears acute tones better than grave. In such a case we may, with great probability, suspect too great tension of the membrana tympani. This circumstance may be of important use in the obscure diagnosis of diseases of the ear." We would hint to Professor Müller to pay a visit to his townsman, Dr. Kramer, who, we are persuaded, would make the Professor acquainted with phenomena, of which he appears at present not to have the slightest idea. We do not mean to deny that tension of the membrana tympani has great influence over the power of hearing, but we assert, that any increased tension that may occur, can never be the sole or even principal cause of a great degree of deafness.

*Mode of propagation of sound to the labyrinth.* Through what medium do the undulations of sound pass from the membrana tympani? In regard to this question, three different views are entertained by physiologists: 1. Some assert that the vibrations of the membrana tympani, excited by sound, are communicated to the ossicles, and through them conducted to the vestibular fenestra. 2. Others suppose the vibrations to be propagated merely through the air in the cavity of the tympanum to the membrane of the fenestra rotunda. 3. Others again admit a combination of both views.

The opinion that the vibrations of the membrana tympani are communicated through the ossicles to the vestibular fenestra is the oldest. First broached by Koyter, as Dr. Lincke informs us, it was objected to by Fabricius ab Aquapendente on the ground that the ossicles do not form a continuous whole. On the same grounds, Treviranus doubted the propagation of sound through the ossicles. And whatever Treviranus asserts, on a subject like this, must command our highest consideration, as he was what few have been or are, at once, an anatomist, a physiologist, and a mathematician.

The second opinion, that the vibrations of the membrana tympani pass through the air in the cavity of the tympanum, and affect the labyrinth by acting on the membrane of the fenestra rotunda, was first enounced by Schelhammer. It was maintained, as we have seen, by Treviranus. Scarpa supposed sound to pass through the fenestra rotunda, but he also admitted that it was propagated by the ossicles to the labyrinth; he must, therefore, be classed among the supporters of the third opinion.

Those who maintain this second opinion limit the use of the ossicles to the putting of the membrana tympani, as also the membrane of the vestibular fenestra, and indirectly the membrane of the fenestra rotunda, together with the membraneous labyrinth, into different states of tension.

The third opinion is the most generally received. A great partiality seems to be entertained for the view that sound is propagated through the chain of small bones. But driven from this exclusive view, by the fact that the membrana tympani may be more or less destroyed, and the malleus and incus lost, without absolute deafness being the consequence, physiologists have taken refuge in the middle view, that sound is conveyed to the labyrinth both by the air of the tympanum and the ossicles.

It is always to be kept in remembrance, that the base of the stapes forms part of the walls of the osseous labyrinth, and that with the loss of it the whole labyrinth would become disorganized by the evacuation of the perilymph, and complete deafness, therefore, would be the consequence.

The persistence of some degree of hearing along with loss, partial or complete, of the membrana tympani, and of the malleus and incus, is an *experimentum crucis*, that sound may be conveyed to the labyrinth by the vibrations of the air alone. On the other hand, an impediment to the free circulation of air in the middle ear, from muculent obstruction, produces often a greater degree of deafness than might have been expected, had sound been propagated principally through the chain of ossicles. The question that remains, therefore, is this: Is the deterioration of hearing, consequent to the loss of the membrana tympani, together with the malleus and incus owing to their no longer conveying vibrations to the labyrinth? Or is it owing to the following circumstances singly or conjointly?—to the air in an injured tympanum, not being in the same condition for propagating sound, as it is when contained in an entire tympanum?—or owing to the organic change in the membrane lining the tympanum, and consequently, that part of it closing the fenestra rotunda and fenestra ovalis, effected by the disease which produced the destruction of the membrana tympani, or by the subsequent exposure?—or owing to the contents of the labyrinth and membrane of the fenestra rotunda being no longer put into the proper degree of tension, which it is admitted to be one office of the ossicles and their muscles to do?

Before leaving this part of our subject, we would touch upon one other point. At p. 576, Dr. Todd says, "the muscular apparatus of the tympanic ossicles receives its nerves partly from the facial and partly from the otic ganglion, thus exhibiting an analogous arrangement to that of the muscular structure of the iris. Such an analogy renders it extremely probable, that the actions of the muscles of the ossicles are excited in a similar way to that in which the iris is prompted to act." At p. 108, Mr. Pilcher says, on the same subject: "The otic ganglion was imagined, by its discoverer, Arnold, to influence those muscles in a manner similar to that which the lenticular exerts upon the iris, and doubtless this is correct." The looseness of this supposed analogy between the membrana tympani, the internus mallei muscle, and otic ganglion on the one hand, and the iris and lenticular ganglion on the other, is exposed by the anatomical fact, that the tensor tympani muscle has been found by Müller, as well as ourselves, to present the same microscopical characters as other animal muscles, and must, therefore, be considered as voluntary. "This," says Müller, "is also confirmed by the origin of the nervus tensor tympani, from the third branch of the trigeminus, viz., from the nervus pterygoideus internus, and the origin of the nervus stapedius from the facial."

The membrana tympani, in as far as regards its anatomy, cannot for a moment be compared to the iris. We admit with Müller that it is probable that the tensor tympani contracts on the occurrence of a very loud sound, much in the same way that the iris and orbicularis palpebrarum contract, in consequence of the impression of a very strong light on the eye. We think that Mr. Wharton Jones, in his parallel between the ear and the eye, (*Cyclopædia of Anatomy and Physiology*, part xv. p. 562,) has determined the real analogies which subsist anatomically between the different parts of the eye and ear. These analogies are boldly but not loosely drawn; and we think we can discover in them views, which taken in combination with other data, and especially those obtained from the observation of the phenomena attending the employment of injections of air into the tympanum, may yet lead to a more correct physiology of hearing; and we cannot but think, that had Professor Müller taken advantage of the experience and labours of Dr. Kramer, his illustrations of the physiology of the ear, drawn from pathology, would have been more to the point.

*Use of the Eustachian tube.* There are several opinions in regard to this point.

1. "One of the oldest opinions," says Lincke, (p. 484,) "as to the use of the Eustachian tube, is that it serves as an excretory duct for the fluid secreted by the mucous membrane of the cavity of the tympanum, seeing, that when this is not carried off, there takes place, in consequence of the increased accumulation, gradual diminution of hearing, or even deafness." This is certainly one use of the Eustachian tube, and a very important one in a practical point of view. Müller thinks that it is by the motions of the vibratile cilia of the mucous surface that the mucus is carried off.

2. Another function of the Eustachian tube, admitted by many excellent physiologists, is that it serves to give vent to some portion of the air in the cavity of the tympanum, when compressed by the tension of the membrana tympani, supposed to take place during a very loud sound.

3. It is agreed that the principal function of the Eustachian tube is to admit the entrance of air, raised by its passage through the nostrils, to the temperature of the body, into the tympanic cavity, so that an equable pressure may be kept up, and the membrana tympani, as well as the air contained in the cavity, (which air must be looked upon as an indispensable component part of the apparatus of hearing,) thus kept in a state to vibrate in the due degree. Müller says that obstruction of the Eustachian tube always causes dulness of hearing and buzzing in the ears. In this assertion, Müller is mistaken; buzzing in the ear is not always an accompaniment of obstruction of the Eustachian tube; and as to dulness of hearing, we have met with a case to show that the power of hearing a watch, at the distance of fifteen feet, is compatible with obstructed Eustachian tubes.

4. It has been supposed, but with no good reason, that the undulations of sound are propagated, not only through the external auditory passage, but also through the Eustachian tube, into the cavity of the tympanum. With no better reason it has been said, that the sound of one's own voice is carried through the Eustachian tube to the proper organ of hearing, whilst the external auditory passage served to convey every other sound.

*Labyrinth.* The labyrinth is the organ of hearing. As, on entering the eyeball, light has new media to traverse, so, on entering the labyrinth, new media present themselves for the transmission of the undulations of sound to the nervous expansion. The transmitting media of the eyeball have *shapes*, &c., adapted to the purpose they have to effect. We cannot but suppose that the transmitting media of the labyrinth and the passages which contain them are shaped &c. conformably to the laws regulating the transmission and concentration of sound.

*Functions of the auditory nerve in the labyrinth.* According to the experiments of Flourens on pigeons, the nervous substance expanded in the vestibule is the essential part of the organ of hearing, and all other parts may be removed without the sense being thereby completely annihilated.

In regard to these experiments, Dr. Todd remarks (p. 571), "One can scarcely imagine vivisections less likely to lead to useful results than those which involve the exposure of the deep-seated internal parts of the ear, a dissection which, even on the dead subject, demands no ordinary skill; nor can we refrain from expressing our opinion that, had M. Flourens never attempted these experiments, physiology would have been none the worse, and our respect for his humanity would have been all the greater." We do not look upon the results of Flourens' experiments of such inconsiderable value as is here expressed. It requires no great skill, nor any great disturbance of the parts to expose the labyrinth in pigeons. In regard to the inhumanity of the proceeding, the question resolves itself into this: Are vivisections allowable at all? While we answer, "only under circumstances, when the point to be determined appears to be, according to the calculation of probabilities, within our reach," we would say, that M. Flourens has not, in the case under consideration, exceeded the just limits.

As to the part which the semicircular canals play, many hypotheses have been broached, but nothing is really known.

*Cochlea.* Weber considers it probable that the undulations of sound, propagated through the bony substance of the skull to the organ of hearing, especially act on the nerves of the cochlea, and that, on the contrary, the undulations of sound, received from the external air, through the membrana tympani, especially act on the membraneous labyrinth. In regard to this opinion of Weber, Dr. Todd says, the following considerations favour these views:

"It is an admitted fact in acoustics, that sounds are most perfectly conducted by substances of uniform elasticity, and that when propagated from air or water to a solid, or from a solid to air or water, they are conducted much less completely. Now, inasmuch as the cochlea may be regarded as part and parcel of the cranial bones, the sounds which are propagated by these bones, would reach the nervous expansion in that portion of the labyrinth, by the most direct route; whereas, to affect the remaining parts of the labyrinth, the sound must be conducted from the bone through the perilymph to the membraneous vestibule and semicircular canals. Moreover, when it is considered that the cochlear nerves are disposed in a radiated manner in the lamina spiralis, it will appear evident that the oscillations propagated to the petrous portion of the temporal bone, must exert a direct influence on the cochlear portion of the auditory nerve." (*Cyclopædia*, No. xv.)

All this reasoning is founded on the assumption, that the cochlear nerve is as much the nerve of hearing as the vestibular nerve. Comparative anatomy, and the experiments of Flourens, so much underrated by Dr. Todd, teach us that neither it nor the cochlea is essential to hearing. And we would humbly submit, for the consideration of physiologists, the following peculiarities of the cochlear nerve, and then ask (with Mr. Wharton Jones), whether the cochlear nerve is the same in function with the vestibular?

In the first place, the cochlear nerve appears to differ somewhat, in its microscopical structure, from the vestibular nerve; and in the second place, it is distributed in a stratum of the body quite different from the vestibular nerve—quite different from the retina, and, indeed, different from any other nerve of sense.

At p. 528, Dr. Lincke says: "If we consider more closely what has been said regarding the cochlea, we cannot be inclined to view it as an organ of particular tones, whether musical or articulate; and the less so, as the distinction of different objects is a purely psychical function, and, therefore, not to be looked for in the individual parts of an organ of sense. Hence, nothing further remains for us but to recognize (with Scarpa, Joh. Müller, Esser, and some others,) in the cochlea an apparatus, intended to present, in the smallest space, a large surface for the expansion of the nerve." This may be all true, but the question still remains, "what is the function of the cochlear nerve?" Moreover, the shape which the perilymph, contained in the cochlea, receives from the latter is not explained.

In considering the function of the waters of the labyrinth, it is essential to keep in view the shape they acquire from the cavities containing them.

We shall close this part of our subject with a few short analytical extracts from the physiological considerations with which M. Breschet follows up his description of the structure of the labyrinth.

"The membraneous walls of the semicircular tubes, of the middle sinus, and of the sac, held suspended between two liquids, are in the most favorable condition for receiving and transmitting the sonorous undulations; the sac, the middle sinus, and the semicircular tubes are composed of a tissue, of a nature between the membraneous tissue properly so called and cartilaginous substance. Hence, these parts are endowed with such elasticity and resistance, that the walls of the canals do not fall together when the liquid they contain is evacuated.

"The properties of tissue must be of a high degree of importance in the functions of these organs; for, on the degree of elasticity and rigidity of the walls of the semicircular tubes, of the sac and middle sinus, placed as they are in the midst of a fluid, must depend the degree of sensibility of the organ."

"If the description of the vestibule, of the two liquids, of the membraneous pouches, and of the nerves which terminate at them, has been well comprehended, we are naturally led to infer that the sonorous undulations can arrive at the expanded branches of the acoustic nerve only by the intermedium of liquid strata, and that in fact these nerves are placed between two distinct strata of liquid. The first liquid is situated between the osseous walls of the labyrinth covered by their periosteum, and the membraneous labyrinth, and in the cochlea. The second is contained in the semicircular tubes, the middle sinus, and the sac.

"The small disc of the stapes corresponding to the vestibular fenestra, instead of transmitting directly the sonorous undulations to the acoustic nervous filaments, which are expanded on the membrane forming the semicircular tubes, the middle sinus and the sac, transmits them only to the *perilymph*. The *endolymph* has not only for its function to concur in maintaining the membraneous walls of these tubes in the best condition for the reception and transmission of the sonorous vibrations, but it also holds in suspension lapilliform concretions, or a pulverulent matter, with which the extremities of the nerves correspond. . . . . I presume that the otolites and otoconies have for their use to communicate to the nervous extremities a more vivid and energetic impression than a simple liquid like the endolymph could do; for the vibrations of a solid body are much more sensible for their force and degree of intensity than those of a liquid body." (*Breschet.*)

### III. DISEASES OF THE EAR.

The history of the progress of ear-medicine we have sketched on a former occasion. (*Br. and For. Med. Rev.* vol. III. p. 79.) Here we would make only a few supplementary remarks. In consequence of the deep and concealed situation of the principal parts of the organ of hearing, it is very difficult to investigate its diseases properly; indeed, it has been looked upon by most surgeons as a thing impossible; and they have, therefore, with few exceptions, hopelessly abstained from making any attempts to overcome the difficulties. It would have been a sad thing if the progress of medicine, which has opened up to us new modes of investigating the diseases of other organs equally concealed and deep seated as the ear, should have failed to shed some light on its pathology. Happily this is not entirely the case, although the amelioration has been slow and partial. Now, thanks to the labours of Itard, Deleau, and Kramer, we are enabled to explore the diseased states of the ear pretty successfully.

As, in treating diseases of the ear, our efforts are in too many cases only partially successful in restoring hearing, as there is little opportunity for a display of dexterity in operating, and as no brilliant results are obtained, like those from tying an artery for the cure of aneurism, cutting for the stone, or extracting a cataract, there has been little inducement to the ambitious surgeon to bestow even a passing glance on the subject. The consequence has been, that the practice in diseases of the ear has been left almost entirely in the hands of persons unprepared by education for the exercise of a scientific profession. Hence the name *aurist*, which has been employed to designate a person who professes to cure diseases of the ear, is in some degree synonymous with *quacksalver*, and that, perhaps, even more than the names *oculist*, *rupture-doctor*, *bone-setter*, and the like.

Regular medical men do not disdain to operate for hernia, reduce a luxation, and even couch a cataract, or lay open a fistula *in ano*; but it would appear that there is something so peculiarly repulsive about that unfortunate organ, the ear, as to render it unworthy of such favour at the hands of the profession. Almost all seem to shrink at the idea of having anything to do with it, lest they should be put down as *aurists*. They are right, if an attempt to treat the diseases of the ear must necessarily make them act the part of the quack; but very wrong if the practice of ear-medicine involve no such alternative. And wherefore, let it be asked, should it do so? The ear is capable of being studied anatomically, and why not also surgically and medically? If, by doing so, we shall not always be able to cure, we shall at least discover how far the interference of art is capable of being of service: and that in medical matters, we hold to be great knowledge. Is not the ear as noble an organ as the eye? And are these two admirable organs of sense of so little consequence to the moral, intellectual, and physical man, as to deserve less consideration from the regular surgeon than a leg or an arm?

It is worthy of remark that, in the schools of medicine, great pains are taken in giving instruction on the subjects of hernia and lithotomy; and it is a *sine qua non* with the young man, going in for examination, to be able to describe exactly the inguinal and crural canals, the position and connexions of the urethra and prostate gland, and, above all, the origin and course of the epigastric and pudic arteries; and all this in reference to operations which not one surgeon in twenty is ever called on to perform; whilst diseases of the eye and ear, which are always occurring in the daily routine of general practice, are passed over, in the course of general medical and surgical instruction, commonly with a very superficial and inadequate notice. Far be it from us to imply by this, that hernia, lithotomy, or any other great operation in surgery, should be less studied than it is; but surely, while the one is done, the other need not be left undone.

Having, in our notice of Dr. Kramer's work, already referred to, considered pretty fully the diseases of the ear in general, we shall, on this occasion, confine our attention to one part of the subject only, in order that we may be able to discuss it the more fully. We do this the rather, as our knowledge of the diseases of the ear is not yet so far advanced as to allow us to pronounce a general summary judgment on any work on the subject. All that can properly be done is to examine some one of the principal points in detail.

The point we mean to take up is the affections of the middle ear; these being, perhaps, when compared with the affections of other parts of the body, the most peculiar, both in their diagnosis and treatment; a knowledge of them, moreover, being of paramount importance, for a perfectly just comprehension of the other diseases of the organ.

As being indispensable for the diagnosis and treatment of the diseases of the middle ear directly, and indirectly as a means of diagnosis at least in many kinds of deafness, catheterism of the Eustachian tube, and the employment of injections of atmospherical air, will, of course, fall to be particularly considered. The books before us, which will serve as a text, are principally Pilcher's and Deleau's.

Under the head of diseases of the middle ear, Dr. Kramer admits:

1st. Inflammation of the mucous membrane of the middle ear: *a*, with accumulation of mucus; *b*, with stricture of the Eustachian tube; *c*, with obliteration of the Eustachian tube.

2dly. Inflammation of the cellular tissue and of the periosteum in the cavity of the tympanum or true otitis interna. This occurs in two forms; *a*, the acute, and *b*, the chronic.

Before going further, we beg the reader to take up Kramer's work, and read over what is said on the above subjects, as the proper understanding of them is of immense consequence in ear-medicine; and as the criticism and counter-criticism, into which we are about to enter, bear particularly on the point.

In the first place, then, the reader will remember that we have already remarked, in the anatomical section of this article, that the lining membrane of the cavity of the tympanum is fibro-mucous—at once a mucous membrane and periosteum. This membrane may be the seat of simple blenorrhœal inflammation, and it may also be the seat of a more violent inflammation, of what is called *otitis interna*. In the former case there is an undue secretion of mucus, which, accumulating, becomes a cause of deafness, by impeding the entrance of air, and obstructing the free movements of the parts contained in the cavity of the tympanum. In the latter case, the bony walls of the tympanum sooner or later participate in the inflammation and its consequences.

It may, perhaps, be allowable to speak of the first inflammation, as "inflammation of the lining membrane of the cavity of the tympanum in its capacity of mucous membrane;" and the second, as "inflammation of the same membrane in its capacity of periosteum." Kramer describes otitis interna as "inflammation of the cellular tissue and of the periosteum of the cavity of the tympanum;" but, as there is scarcely any cellular tissue, and as the periosteum is not distinct from the mucous membrane, ours is the more correct pathology. With these remarks, we beg to say that our experience leads us to agree generally with Kramer.

Not so Mr. Pilcher:

"That acute inflammation of the tympanal cavity occurs, commencing ordinarily in its mucous membrane, and extending to the other structures, almost every day's experience convinces us. Dr. Kramer has arranged it under two heads, that of inflammation of the mucous membrane, with mucous accumulation, and that of the sub-mucous cellular tissue; which arrangement corresponds to Itard's *catarrhal and purulent internal otitis.* This division is with difficulty recognized in the majority of acute cases, and experience leads the author to justify the criticism of M. Itard's reviewer in the Edinburgh Journal." (*Pilcher*, p. 179.)

The criticism in the Edinburgh Medical and Surgical Journal, here alluded to by Mr. Pilcher, is the following:

"Although our author has divided the internal otitis into catarrhal and purulent, we must acknowledge our inability to discover the difference, either from his account of the symptoms, or from the little which we have had occasion to know personally of the disease. We conceive it, indeed, essentially irrational to attempt to establish a distinction of this kind, in a morbid state of a part which must be the same in the beginning and through the course of the disease, and seems to differ only in the degree to which it may proceed. Our author, indeed, himself seems to overlook the difference, unless in the termination, and, if we understand him aright, makes one history of symptoms serve for both varieties." (*Ed. Journal*, vol. xix., p. 90.)

This criticism of the Edinburgh reviewer is correct, as regards the object it was written on; but, in applying it to Kramer, Mr. Pilcher loses sight of the fact that, concerning Itard's division, Kramer expresses himself to the following effect:*

"Itard establishes two forms, *otite interne catarrhale* and *otite interne purulente*, quite correctly as to name, but incorrectly according to his description. Thus, according to Itard's account, the difference between the two lies only in the different degree of severity of the symptoms, but no difference in regard to internal character is stated, though such a one really exists between catarrhal and phlegmonous inflammation. Itard's *otite interne catarrhale* is said, in by far the greater number of cases, to end in perforation of the membrana tympani and evacuation of the matter in this way, because the Eustachian tube has become closed by the inflammation;—an altogether erroneous opinion, as in perforation of the membrane of the tympanum, which always takes place in consequence of inflammation of it, the Eustachian tube is generally found open, thus, the escape of the matter through it would not have been stopped, if an accumulation had really existed in the cavity of the tympanum. On the other hand, we have observed many very inveterate catarrhal inflammations of the middle ear, with actual obstruction of the Eustachian tube, without any of the violent symptoms attributed to them by Itard, and without destruction of the membrana tympani having ensued, even when the case had been treated with the greatest neglect. Lastly, Itard cannot make good his opinion, because, in the cases which he relates as examples of his *otite purulente interne*, he did not examine either the perforated membrana tympani or the cavity of the tympanum, which he pretends to have been the seat of the suppuration, or the Eustachian tube, the obstruction of which he presupposes."

From this extract we find that, substantially, Kramer confirms the justice of the strictures on Itard's division of otitis, contained in the Edinburgh Journal, but at the same time vindicates the correctness of his own admission of a distinct *catarrhal otitis*.

In regard to the doubts expressed by the reviewer as to a *catarrhal* or *blenorrhœal otitis*, distinct from a *purulent otitis*, it is to be remarked,

* We quote from the original, not having at hand Dr. Bennett's accurate English version of the work.—Rev.

that he does not appear to have employed catheterism and injection of the Eustachian tube, and could, therefore, know nothing practically of any such distinction; particularly, as in the former, the inflammatory symptoms are very seldom considerable, as, in fact, the purely inflammatory stage is not unfrequently overlooked. Hence, as Dr. Kramer says, the catarrhal otitis is in general indicated by no other subjective symptom but deafness; and it is only by means of catheterism and injection that its diagnosis can be established. We shall see that Deleau gives the same view of the matter.

The excuse we have made for the Edinburgh reviewer can hardly be extended to Mr. Pilcher, as he is accustomed to the employment of catheterism and injection of the Eustachian tube. He appears to confound Kramer's catarrhal otitis with Itard's, notwithstanding the care Kramer has taken to point out the difference.

Mr. Pilcher's description of true phlegmonous *otitis interna* is illustrated by some very valuable cases, chiefly "supplied by friends to the essayist." Mr. P. discusses, at one part of his work, acute otitis interna, and at another part the chronic otitis interna, not a very good plan, as the two forms of disease are not always independent of each other. This is seen from the cases themselves, for all those which Mr. P. relates, in illustration of acute otitis interna, are cases of the acute disease supervening on a chronic one. The best marked case of idiopathic acute *otitis interna*, in Mr. Pilcher's book, is given at p. 165, under the head of *otitis externa.* It is adduced as "a case which points out the great destruction in which the ear may be involved from surrounding disease." Mr. P. further remarks, "it appears that in this case an abscess originated in the cellular tissue, between the meatus and parotid gland, which, in its extension ulcerated into the auditory canal, reached the cavity of the tympanum, and eventually the brain, the pressure upon the nerves in the neighbourhood having interrupted their function."

We doubt this pathology; and think that if any one will read carefully the case he will come to the same conclusion as ourselves regarding it, viz., that it was an inflammation and abscess of the tympanum; that the abscess burst through the membrana tympani, and also pointed at the mastoid process; that the petrous bone and aqueduct of Fallopius, and, of course, the portio dura, became involved in the disease; and, lastly, that inflammation extended to the brain or its membranes.

We now come to consider some details in Mr. Pilcher's account of catheterism of the Eustachian tube.

"Both authors, Itard and Kramer, propose to sound the tube, by means of a catgut run through the instrument; for which purpose, and particularly to inject fluids into the tympanum, it is necessary to fix the catheter by a frontal bandage, which is furnished with a pair of strong holders or forceps. The author, when he first practised this operation, was desirous to dilate a contracted tube, and, therefore, gave to his instrument a longer and more gradual curve; the bend near the handle allows it to rest more conveniently against the tip of the nose. This shape was determined upon, after trying a wire, bent in various degrees, upon a preparation of a perpendicular section of the head and face, when that depicted in the sketch, (plate xiv. fig. 4,) was found to enter the tube, and to run along nearly an inch of its extent with great ease. A graduated silver-wire stilette is also appended to it, which may be introduced beyond the catheter, if the surgeon should deem it advisable, or a catgut string may be used, which, at the same time that it is safer, will answer the purpose as well; and the author has lately used stilettes of whalebone slightly enlarged at the point, which he finds much more convenient and useful than when made of other materials." (*Pilcher*, p. 304.)

"When used for the purpose of investigation, the wire may be carried further onwards, or, what will be much the safer, the catgut or whalebone sound may be passed through the catheter. If it should reach the tympanal cavity, it will give rise to pain, often severe, to a loud cracking noisy sensation, extending to the mastoid cells; these feelings will be more marked in proportion to the healthy state of the tympanum. The surgeon must be especially careful not to injure the ossicula, the avoidance of which will require great caution, passing, as they do, across the cavity; the stilette must, therefore, just reach the tympanum without entering it." (*Ib.* p. 306.)

It appears to us that Mr. Pilcher is rather free in passing sounds through the Eustachian tube into the tympanum. Cases in which it is really indicated are comparatively rare; and how little benefit is to be obtained from it, even in those cases, may be learned by referring to Kramer or Deleau. We hold it to be a very unnecessary and reprehensible proceeding to push a probe, whether it be a metal or a whalebone stilette or a catgut string, along the Eustachian tube into the tympanum, until a well-directed examination, by means of streams of air, directed from an air-press, along the Eustachian tube, has unequivocally proved the existence of a stricture. A sound can never afford the same information, regarding the state of the middle ear, as the air-douche.

"Deleau," continues Mr. Pilcher, "introduced the air-douche for the purpose of removing matter from the cavity, and also distending the contracted tube, which Kramer considers a grêat improvement upon the injecting of fluids. Both these intelligent aurists used an air-press, for the purpose of increasing and regulating the force employed. The author, however, daily experiencing the great facility with which air and fluids may be introduced, both as to quantity and force, by means of a common syringe, accurately fitted to the catheter, does not hesitate to declare his conviction, that the ceremony and inconvenience of the air-press may be dispensed with." (p. 307.)

We cannot but admire the boldness which dictated this, unsupported, as the assertion here is, by the history of a single case effectually treated, and gainsayed, as it is, by the experience of Deleau, Kramer, and, we would add, of ourselves.

"It is of course requisite," continues Mr. Pilcher, "that the operator should steady the instrument with his left hand, while using the syringe with his right." To say nothing of the pain unnecessarily inflicted on the patient by this means; we would ask how, in this way, does Mr. Pilcher listen to the sounds produced, supposing any be produced, by such a feeble stream of air? Does Mr. P. venture to inject air without listening to the sounds produced? If Mr. P. had ever used the air-douche properly, as a means of exploring the state of the middle ear, we are confident he would not have spoken so heroically of sounding with stilettes.

Mr. P., it is seen, says the syringe must be *accurately fitted to the catheter*. On this point we feel called upon to give a practical caution.

Not that we dread much harm from the feeble stream of air, projected from Mr. P.'s *common syringe*, whilst the nozzle is "accurately fitted to the catheter," but lest any one, having an air-press, should suppose it necessary, as we have observed it to be the common impression, to push home the nozzle of the tube of the air-press into the dilated end of the catheter. The nozzle of the tube of the air-press should be held, during the operation, so loosely in the dilated end of the catheter, that there may be room for air to regurgitate. If the nozzle of the tube of the air-press ought not to be pushed home into the catheter, much less should the point of the catheter be pushed far up into the Eustachian tube. If it were so, and if a stream, necessary to overcome an obstruction, were injected, what would be the result if no room were left for regurgitation? We shall answer this question in our analysis of Deleau's work.

Obstruction of the Eustachian tube is not removed by the mere force of the air-douche, but by the continued battering of the stream breaking up the obstructing mucus, and exciting the mucous membrane to increased secretion, for the time by which the thickened mucus is softened, and thus disposed to be more readily dispersed at subsequent operations by the air-douche.

Dr. Deleau is distinguished for having introduced the injection of streams of atmospherical air through the Eustachian tube into the tympanum as a means of diagnosis and treatment in the diseases of the ear. We have, in a former Number, alluded to the claims which might be set up in favour of our countryman Cleland as the first proposer of injections of air into the middle ear. It is nevertheless but justice to concede that all that has been awarded to Deleau, whether in the way of honorary or more substantial testimonials, has been fully deserved by him. We, however, confine our eulogium to the simple circumstance of introducing the air-douche. We cannot join the reporters of the Institute in their unqualified approbation of other details in Deleau's practice, such as the preference he gives to gum-elastic catheters. We entirely concur with Dr. Kramer in his remarks on Dr. Deleau's frivolous objections to metallic catheters.

The first Report of the Institute on Deleau's labours is dated Dec. 19th, 1822. At this time Deleau had not thought of the air-douche; he merely employed catheterism of the Eustachian tube and liquid injections, and that in a manner not at all superior to what had been done before in this country; and yet the labours of Cleland, Douglas, and Wathen are very unjustly and incorrectly stated in the report. They are in fact stated in a way which shows that the reporters had not taken pains to inform themselves on the subject, but hastily took it upon them to give an opinion in a matter they were quite ignorant of. We, however, fully and heartily concur in the following judgment delivered by Savart, in the Report No. 4, dated 7th Dec. 1829:

"In short it appears to us that M. Deleau has done real service to the medical art by his ingenious invention of injections of air for the purposes of forming the diagnosis and prognosis of affections of the middle ear. We, therefore, consider his work deserving the approbation of the Academy."

Though Dr. Deleau's work abounds in verbiage, it contains many shrewd remarks, and shows that the author is a man of exquisite practical tact—a man who might have written a more generally useful book, if he had condescended to enter more into descriptive details. In regard to his cases, we would hint, that they are not related with all the definiteness and precision desirable.

The first chapter of the work contains a physiological enquiry into the part which the atmospherical air contained in the middle ear plays in the process of hearing. The author insists, correctly, that it forms a necessary component part of the apparatus of the sense.

"Before employing," says Deleau, "in the diagnosis and treatment of the diseases of the ear, pure air at the temperature of an inhabited room, I had tried air charged with emollient or aromatic vapours: but whatever the qualities were which it acquired in traversing a reservoir of liquid, there almost always resulted an augmentation of the deafness; and it frequently happened that the medication which I endeavoured to apply to the affected organ, gave a result contrary to my expectations. The vapours which I judged sedative, and which are so for other organs, excited pains and often inflammation. Hence, experience has led me to the almost exclusive employment of douches of dry air. (*Deleau*, p. 46.)

"After having studied the action of the air on the middle ear in the natural state, we proceed to examine what is the mode of action of the lesions of the organ on the presence and on the circulation of this fluid in the Eustachian tubes, the cavity of the tympanum, and the mastoid cells. The diseases of the pharynx which are communicated to the mouth of the Eustachian tube, and contract and alter it, shall first fix our attention. We shall then examine the numerous lesions of the middle of this canal: these will conduct us to the exploration of muculent accumulations in the cavity of the tympanum. Lastly, we shall see that the perforation of the membrana tympani is far from being advantageous to the circulation of air in the apparatus of hearing." (*Ib.* p. 62.)

All these are very important points in the study of the diseases of the ear; we therefore think it necessary to devote a page or two to their consideration.

At p. 44, Deleau says, "Surgeons occupied in treating cases of cleft palate must have met with many deaf persons among those afflicted with this congenital infirmity. No one has made mention of the fact." We would remind M. Deleau that Sir Astley Cooper relates in his paper on perforation of the membrana tympani, in the Philosophical Transactions for 1801, (vol. xci. p. 443,) the case of Mr. Round who had a congenital affection of the palate so that he could not blow his nose; this case by Sir A. Cooper is related incidentally; but Dr. Dieffenbach of Berlin has dwelt professedly on the fact in a paragraph in his Chirurgische Erfahrungen, dritte und vierte Sammlung, (Berlin, 1834, § 205, pp. 261, 262,) under the title "Ueber eine bis jetzt nicht erkannte Ursache der Taubheit."

Deleau attributes the deafness in such persons to a dryness and almost always a chronic inflammation of the mucous membrane of the throat, in consequence of its exposure to the contact of the cold atmospherical air, which chronic inflammation extends to the middle ear. Dieffenbach says, "I am of opinion that the cause of deafness in persons with cleft palate is a closure of the Eustachian tube. By the fissure of the palate, the lips of the guttural orifice of this passage are so much relaxed that they lie upon each other and hinder the free entrance of the air to the inner ear. . . . . Staphyloraphy always completely removed this. When the operation was successful there occurred in some cases at first a sensitive fineness of hearing, which afterwards gradually subsided."

Dieffenbach's explanation of the proximate cause of deafness occurring along with cleft palate may be in some degree applicable, but we are inclined to attribute the principal share to the causes mentioned by Deleau.

In regard to the influence of tumefaction and induration of the tonsils on hearing, Deleau remarks as follows:

"The tonsils, when they become double, triple, or quadruple the usual size, by the action of the causes which give rise to inflammation of the throat, have not in general, by reason of their development, a prejudicial action on the mouth of the Eustachian tube, and consequently on the entrance and exit of air in the middle ear. I have met with tumefactions so great that they threatened the life of the individuals, without causing the slightest deafness. . . . . . It is rather when these glands are subject to pass into the state of acute inflammation, or when they are surrounded by a red and tumefied circle which invades the lateral walls of the pharynx, that hardness of hearing is perceived, or noises in the ear which the patients compare to the boiling of water, or to the rustling of leaves shaken by the wind. A condition still more serious which these glandular bodies take on, is their development from before backwards, so as to separate the pillars of the fauces. Hardness of hearing almost always accompanies flattened glands which tend rather to sink into the flesh than to project into the throat." (*Deleau,* p. 70.)

The following is Deleau's remarks on the inflammation of the lining membrane of the middle ear, to which we have in a preceding page referred.

1. As to accumulation of mucus, he says, "the mucous membrane, the seat of this too abundant secretion, rarely passes into the acute phlegmonous inflammation, called *catarrhe douloureux de l'oreille.* The only signs of this kind of lesion are drawn from the variations of the hearing, which are pretty frequent." (p. 76.)

2. On strictures of the Eustachian tube, our author thus expresses himself:

"Strictures of the Eustachian tube, accompanied by sub-acute inflammation, are principally met with in individuals of a sanguineous temperament, subject to frequent attacks of otitis. Frequently, however, the patients do not remember having experienced the slightest pain ; it is, then, that the inflammation is developed in an insensible manner, has passed into the inflammatory state, and has degenerated into stricture without the manifestation of any other symptoms than diminution of hearing; I have often seen such cases confounded with affections of the auditory nerves. . . . . It is true that they are appreciable only by means of catheterism and the air-douche. . . . The treatment by sounds ought to be seconded by the most active derivatives; frequently the success corresponds neither to the patience of the patients nor to the sagacity of the practitioner." (*Ib.* p. 78.)

3. *Chronic inflammation and muculent obstruction of the cavity of the tympanum.*

"It may readily be conceived, without any great knowledge of the pathology of the ear, that the privation of atmospherical air, in those cases of inflammation of the tympanic walls, is rarely the only proximate cause of the deafness, for the tissues through which the sonorous undulations have to pass, have acquired a degree of sensibility so great, that the least sound is insupportable. In other cases, this vital property is almost annihilated, either in consequence of thickening of the membranes, or by the compression resulting from effusions into the tympanum." (*Ib.* p. 87.)

We might add, the mere change in the common physical properties of the membrane lining the cavity of the tympanum, and, consequently, of the membranes closing the fenestræ, appears to be a very likely proximate cause of deafness.

*Diseases of the membrana tympani, in which the air-douche is required.* Kramer says, on this subject, that in ulceration and perforation of the membrana tympani, the Eustachian tube is generally open.

This, however, will not be found always the case. But whether it is or no, it might be supposed indifferent, as the air enters the tympanum by the perforation in the membrane. The experience of Deleau, however, proves the contrary ; for, in cases of the sort, he has found "immediately that the obstruction of the Eustachian tube was removed, and the air permeated freely the whole middle and external ear," the hearing distance was considerably raised.

Deleau's third chapter is entitled "*Of the insufficiency of former modes of diagnosis and treatment in diseases of the middle ear.*"

"In the first chapter," he says, "I have studied the functions of the atmospherical air in its relations with the solid parts of the ear; I have demonstrated that it must, as well as they, possess certain qualities, which I have called physiological, else it injures the tissues with which it comes in contact, and it is little adapted to the exercise of hearing. Viewed under this aspect, in its relations with the ear, this fluid ought to be submitted, more than has been hitherto done, to the investigations not only of physiologists but also of anatomists. Do the latter not examine whether the labyrinthic canals are filled with a liquid or gaseous fluid ? Do they not strive to guess the composition of the fluid ? Would it not be useful for them to know whether it is transported from a reservoir to a canal, or from one canal to another, during the succussion which the whole organ receives from the sounds which surround us ? Yes, truly: why then have they neglected to study the presence, the movements, the qualities of the air introduced into the middle ear?" (*Deleau*, p. 97.)

Our author goes on to say that in his second chapter he has considered the morbid states of the middle ear, which impede the free circulation of air; but he says his opportunities for making *post-mortem* examinations have not been so great as those of his "honorables confrères."

"However," he continues, "I have succeeded in exploring this organ better than has been done before, by modifying the exploring instruments of my predecessors ; . . . . and especially employing, as a principal exploratory means, a fluid, set in motion in the middle ear, indicating, by the sounds there developed, whether the passages be free, contracted, or obstructed ; I have found in flexible instruments of extreme simplicity, in the address of a hand endowed with a delicate sense of touch; and in the observations made by my ear applied to that of the patient, wherewith to make up for the sight, which in all surgical operations is the most certain guide of the operator." (*Ib.* p. 99.)

The following observations, in reference to the peculiarities of the affections of particular organs and treatment on general principles alone, are important.

"Could they not comprehend that the conformation alone of certain organs gives origin to lesions which cannot be met with in any other part? Could they forget that special functions are found impeded, annihilated by their products, or by the presence or absence of the bodies which put them into play ? Ought a stone in the bladder, a flattened auditory canal, an Eustachian tube, which has lost its conformation from an inflammation, long ago subdued, be treated by setons and moxas ? If you see in these maladies only retention of urine in the one case, and deafness in the other, leave the treatment of them to more experienced hands, or, at least, listen to and follow their advice. If vision could not be exercised for want of light, would you seek to apply your medicaments to the eyeball? If air is not introduced into the middle ear, how could you succeed in making the sound arrive at the labyrinth ? Remove the obstacles to the introduction of this air, indispensable to hearing, and leave at rest the organic tissues when they are not diseased. What would you think of a practitioner who should insert a seton in the neck, or who should purge every two or three days, in order to remove an accumulation of cerumen in the auditory passage ? Surely you would blame his conduct. Permit me, then, to do the same, every time you treat, in a similar way, an obstruction, quite as palpable, in the Eustachian tube." (*Deleau*, p. 109.)

*Diagnosis.* "I now proceed to point out the effects of douches of air on the middle ear in the state of disease, and enumerate the sounds which they produce according as this or that part of the organ is affected. This simple exposition will serve as an introduction to the chapter dedicated to the diagnosis. The ***bruit de pluie***, produced by a moderate douche, is sometimes accompanied or followed by a slight pain and momentary increase of the dulness of hearing. It indicates an exaltation of sensibility—it is a commencement of inflammation. This state can only be recognized and well appreciated when one is certain of having performed, without hesitation and fumbling, the operation of catheterism; for it is easy to suppose that an awkward hand might, by throwing in the air with too great force, give rise to this morbid irritation in the ear; in the same way that too strong light would over-irritate the retina predisposed to inflammation by a delicate operation." (*Ib.* p. 133.)

The condition of the ear, above described, in which Deleau's *rain-sound* occurs, it will be seen, is the same as Kramer's nervous deafness; of which more anon.

"Sometimes," continues Deleau, "the catheter is engaged in the tube some lines only; if we do not take precautions in withdrawing the stilette, the end of the catheter escapes into the pharynx. In this case the air which serves for the douche is dispersed in the pharynx; if the catheter remains in the Eustachian tube, the air regurgitates immediately after having arrived at the extremity of the catheter, and makes the lips of the guttural orifice of the Eustachian tube vibrate. I call the sound which is heard, and which is more or less mucous, *bruit du pavillon.* When it is simple, it indicates a stricture or a complete obstruction, situated in the guttural half of the tube. When the obstacle to the introduction of air into the tympanum is near that cavity, we perceive, on applying our ear to that of the patient, the *bruit de la trompe.* If the douche is given at the pressure of about one atmosphere, both the *bruit du pavillon* and *bruit de la trompe* will be heard simultaneously. The *bruit de la caisse* is more or less mucous; it is general or confined to one point of the tympanic wall. Little practice is required to appreciate the different characters of it." (*Ib.* p. 134.)

Besides the above sounds there are also those of the membrana tympani, which are named by our author *éclats* and *sifflements.*

In chapter vi., which is devoted to the diagnosis of the diseases of the middle ear, Deleau gives (p. 144) the following ***Table of the known lesions of the middle ear, which occasion deafness.***

FIRST CLASS.

| | |
|---|---|
| Affections of the guttural orifice of the Eustachian tube, from disease of the pharynx. | *First Order.*<br>Chronic inflammation.<br>*Second Order.*<br>Tumefied and indurated tonsils. |

SECOND CLASS.

| | |
|---|---|
| Diseases of the Eustachian tube. | *First Order.*<br>Simple obstruction.<br>*Second Order.*<br>Chronic inflammation, { without secretion. / with secretion.<br>*Third Order.*<br>Stricture situated in the { inner half. / outer half. |

THIRD CLASS.

Diseases of the cavity of the tympanum.

- *First Order.* Inflammation without secretion.
- *Second Order.* Muculent accumulation
  - from obstruction of the Eustachian tube.
  - from increased secretion.

FOURTH CLASS.

Diseases of the membrana tympani.

- *First Order.* Inflammation.
- *Second Order.* Perforation.

FIFTH CLASS.

Complications attending disease of the middle ear.

- *First Order.* Combination of the above-mentioned affections.
- *Second Order.* Diseases of the middle and of the external ear.
- *Third Order.* Diseases of the middle ear and of the labyrinth, of the auditory nerves, and of the brain.

*Prognosis.* The subject of prognosis is a very important one in all diseases; but in the case of the eye and ear it is peculiarly so, because a correct prognosis is oftentimes of so much consequence to the worldly concerns of the patient; and the means of giving it correctly are, besides, so much within our reach. Deleau is so fully sensible of this that he commences his chapter on the prognosis of the diseases of the middle ear with the following remarks:

"It is perhaps to the prognosis I give my patients that I owe a part of my practice. Many patients are sent to me by persons whom I had pronounced incurably deaf, but who are not the less ready to recommend me than are those whom I have cured. This is not surprising: for indeed it is as useful to humanity to prognosticate the impossibility of curing certain cases of deafness, and not to subject to treatment the numerous incurables, as to cure those who are curable. Consider how many wounds, bloodlettings, and operations still more painful, one should practise, if he subjected indiscriminately to treatment that immense number of individuals who are constantly running about to consult all the medico-chirurgical notabilities and all the inventors of new nostrums!" (*Deleau*, p. 172.)

M. Deleau's tenth chapter is entitled "Of the accidents sometimes caused by catheterism of the Eustachian tube, and injections of air into the middle ear, and of the relapses which are apt to take place."

The accidents which sometimes occur are: 1. Inflammation of the throat, and catarrh of the tympanum. 2. Emphysema. 3. Rupture of the membrana tympani.

Emphysema has occurred to Deleau six or seven times. Mr. Pilcher mentions that it once occurred to him, and we have heard of other two cases. Kramer says nothing about it; and, of the many times we have catheterized the Eustachian tube, and injected air from an air-press into the tympanum, we have never met with the accident. The emphysema must take place in consequence of laceration of the mucous membrane of the Eustachian tube or posterior nostrils, and will, therefore, be likely to occur when the catheter is either rudely introduced or forced too far into the tube.

Rupture of the membrana tympani is not to be wondered at if the catheter be pushed far up into the Eustachian tube, or the nozzle of the pipe of the air-press pushed home into the dilated end of the catheter, and the air-douche allowed to play with force.

Deleau says, when laceration of the membrana tympani takes place, it is exactly in the middle of its lower half; the opening is round, and is almost always a line in diameter. "It is to be wished," he continues, "that in many cases of deafness, we could produce at will such effects. The patient feels no pain; he does not even know what has happened to him—oftentimes he is agreeably surprised at hearing perfectly well." (p. 330.)

We should not be inclined to perform the operation of perforating the membrana tympani in this roundabout way; this reasoning of Deleau's is something like making a virtue of necessity. Our author continues, "To avoid this rupture during the douche, it is necessary to measure the force of the current, and listen at the ear, to be assured that the column of air returns into the throat with more or less ease." How this is to be accomplished, we have above pointed out, and remarked on the impropriety of pushing the catheter far into the Eustachian tube, so very far as Deleau pretends to do.

On the occasion of our review of Kramer's work, in our Third Volume, we remarked (p. 100), that Mr. Swan wishes to establish that, in a great proportion of habitually deaf people, the auditory nerves are not affected. We stated his opinion to be that, in many such cases, the deafness is owing to a thickened state of the lining membrane of the cavity of the tympanum, involving the small branches of the tympanine nerves; and we still think that this theory of Mr. Swan's accords very well with the good effects derived from Dr. Kramer's practice in nervous deafness; as it appears probable that the ethereal vapours may act directly on the morbid lining membrane.

Dr. Kramer objects to this, saying that it is erecting one hypothesis to support another. We reply that Dr. Kramer has no direct, and but very doubtful inferential evidence, to show that it is the auditory nerve which is affected in those cases which he puts down as nervous deafness. We, therefore, hold that many of his arguments are hypothetical—more so than Swan's, because the opinions of the latter are drawn from actual dissections. Perhaps, however, Swan infers too much from his observations.

In the state of our knowledge of the physiology and pathology of the ear, we can as readily conceive some other part of the organ to be at fault in many of the cases of so-called nervous deafness as the auditory nerves. The enormous proportion of 152 cases of nervous deafness out of 300 cases of diseases of the ear in general, according to Dr. Kramer's own table, staggers us, when we reflect on the comparative rarity of cases of amaurosis.

We think it of immense consequence to the farther progress of ear-medicine to leave the question open as to the condition of the ear in those cases commonly called nervous deafness. We do not deny that many are actually cases of disease of the auditory nerve or brain, but we have no doubt that many are also affections of other structures in the labyrinth, and that some are affections of the membrane lining the cavity of the tympanum, and, consequently, of the membrane closing the fenestra rotunda; and that contributing, with the base of the stapes, to close the fenestra ovalis.

Affection of the membrane lining the cavity of the tympanum may consist in thickening from sub-acute inflammation without increased secretion. Many of the cases related by Deleau as such, seem to be what Kramer would call nervous deafness.

In regard to nervous deafness, Deleau remarks (p. 296), "I cannot comprehend what is meant by paralysis of the auditory nerve, which may, it is said, occur in a child enjoying otherwise perfect health." If what we have above said have any reason in it, it shows the importance of keeping attention fixed on the investigation of this very difficult subject; and it also shows that the injection of ethereal vapours into the tympanum, as recommended by Kramer, good as we can from our own experience acknowledge it to be in some cases, is as yet used only as an empirical remedy.

Deleau's ninth chapter treats of the exploration of the middle ear, and of the treatment of its maladies in persons born deaf and dumb. This is a subject to which our author has devoted great attention. In October, 1837, he communicated to the Academy of Sciences "A General View of his Discoveries in the Semeiology and Therapeutics of the Diseases of the Middle Ear in the Deaf and Dumb." This general view forms the introduction to the work before us, the latter being, he says, the development of the former.

In regard to the treatment of the deaf and dumb, it is to be remarked, that Deleau's cases show that he has, in certain instances, been successful in eliciting some degree of hearing; but in general neither great nor permanent.

We have to find fault with our author for mixing up, as he does, the question of restoring hearing with that of teaching the deaf and dumb to speak. Experience has proved, long ago, that the deaf and dumb may be taught to speak, and even sometimes to carry on conversation without the hearing being in the slightest degree restored. In this case the eye watches the motions of the lips of the speaker, and the deaf person knows from them the words expressed; or, as our old friend J. B., the Chirosophus, will have it, the deaf and dumb person hears with the eye, and thence learns to speak with the tongue.

We fully admit the necessity of *teaching* the deaf and dumb to speak, even supposing hearing enough for the purpose were restored. And if M. Deleau pretended merely, and stated the thing broadly and openly, to teach his pupils to speak, by taking advantage of all possible ways—among others, making them, by surgical treatment, more sensible to the impression of sounds—there would be no room for the objection of which he complains, viz., that "his pupils ought to learn to speak of themselves, without masters and without methods."

Always keeping in mind that, as M. St. Hilaire says, (*Third Report of the Institute on Deleau's Labours*, 23d Oct., 1826,) "M. Deleau a donc ajouté à ses fonctions de médecin celles d'instituteur," we think Dr. Deleau is deserving of all praise for his zealous exertions in the cause of such unfortunate objects as the deaf and dumb.

